# Controlling Virtual Reality With Brain Signals: State of the Art of Using VR-Based Feedback in Neurofeedback Applications

**DOI:** 10.1007/s10484-024-09677-8

**Published:** 2024-11-14

**Authors:** Silvia Erika Kober, Guilherme Wood, Lisa Maria Berger

**Affiliations:** Department of Psychology, University of Graz, Universitaetsplatz 2/III, 8010 Graz, Austria

**Keywords:** Brain-computer interface, Gamification, Neuroenchantment, Neurofeedback, Neurorights, Virtual Reality

## Abstract

The rapid progress of commercial virtual reality (VR) technology, open access to VR development software as well as open-source instructions for creating brain-VR interfaces have increased the number of VR-based neurofeedback (NF) training studies. Controlling a VR environment with brain signals has potential advantages for NF applications. More entertaining, multimodal and adaptive virtual feedback modalities might positively affect subjective user experience and could consequently enhance NF training performance and outcome. Nevertheless, there are certain pitfalls and contraindications that make VR-based NF not suitable for everyone. In the present review, we summarize applications of VR-based NF and discuss positive effects of VR-based NF training as well as contraindications such as cybersickness in VR or age- and sex-related differences. The existing literature implies that VR-based feedback is a promising tool for the improvement of NF training performance. Users generally rate VR-based feedback more positively than traditional 2D feedback, albeit to draw meaningful conclusions and to rule out adverse effects of VR, more research on this topic is necessary. The pace in the development of brain-VR synchronization furthermore necessitates ethical considerations on these technologies.

## Introduction

Virtual reality (VR) technologies enable the creation of customized simulated virtual environments and scenarios that can adapt to the disposition and skills of the users (Kritikos et al., 2021), making learning more accessible and user-centered. In combination with neurofeedback (NF) technology, it is possible to synchronize brain activation states to VR functionalities and environments, enabling the users to interact with the surroundings on a neuronal basis.

NF applications enable a closed-loop system between the brain and a virtual environment. In general, NF systems record changes in brain activation, e.g., by means of electroencephalography (EEG), and process them in real time. Most NF systems use EEG to record changes in brain activation patterns during NF training, given the EEG’s high temporal resolution, allowing the detection of changes in brain activity in the millisecond range. In addition, EEG systems are relatively inexpensive and portable compared to other neuroimaging techniques such as functional magnetic resonance imaging (fMRI) (Sitaram et al., 2017). Hence, we will here focus on EEG-based NF studies.

Specific EEG frequencies typically used in NF training paradigms are associated with specific mental states. For instance, an increase in Alpha waves (~ 8–12 Hz) is associated with a relaxed wakeful state, while an increase in the sensorimotor rhythm (SMR, 12–15 Hz) is observed during a physically relaxed but mentally focused state (Marzbani et al., 2016; Sterman, 1996). Depending on the aim of the NF training, changes in a specific EEG frequency can be fed back to the NF user in real time, e.g., through visual or auditory feedback. Most NF systems use visual feedback, e.g., in the form of two-dimensional objects that change size or move in response to changes in EEG parameters (e.g., Weber et al., 2011). However, changes in brain activation can also be transferred to changes in a VR scenario. Hence, an increase in Alpha activity can let flowers grow in a virtual scenery or an increase in SMR can increase the speed of a virtual space-ship. Through the use of a suitable mental strategy (Davelaar et al., 2018; Kober et al., 2013; Wimmer et al., 2023) to successfully modulate certain EEG parameters during NF training, improvements in the cognitive, motor, behavioral or affective domain are expected to be the result (outcome) after repeated training sessions (Gruzelier, 2014).

**Fig. 1:**
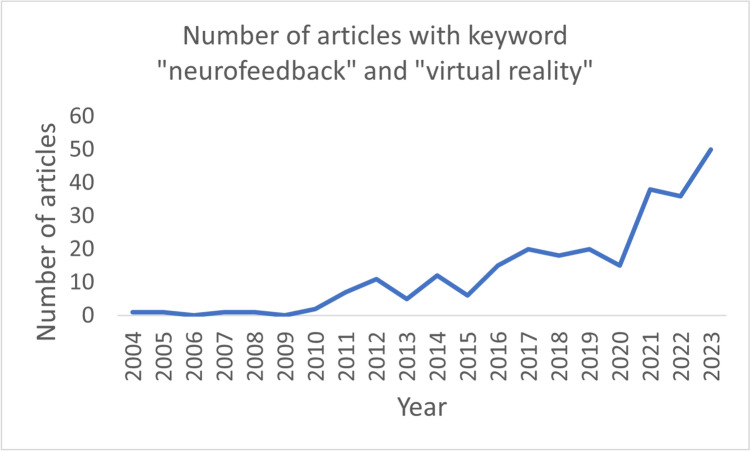
Number of articles per year with the keyword “neurofeedback” and “virtual reality” (Scopus search) from 2003–2023

Game engines make it possible to design virtual worlds relatively quickly and easily without extensive programming knowledge. Recording and processing of brain signals in real time can furthermore be performed through open-source software (Lécuyer, 2016; Prapas et al., 2022; Santamaría-Vázquez et al., 2023; Skola & Liarokapis, 2021). Moreover, the knowledge on how to stream brain data between the recording/preprocessing software and game engines can be acquired through freely accessible online tutorials. Presumably this transformation towards an increasingly easier entry to VR-based NF development has led to a surge in NF studies using VR-based feedback. Figure 1 shows the steady increase in VR-based NF studies over the years (Scopus search, 2003–2023, “neurofeedback” and “virtual reality”). In this context, it is hypothesized that due to the immersive and engaging nature of VR environments, VR-based feedback can improve NF training performance (i.e., successful modulation of target EEG frequencies during NF training) as well as NF training outcomes (i.e., improvements in cognitive, motor, behavioral or affective domains).

The primary aim of this review was to summarize empirical articles that investigated the effects of VR-based NF training on various outcome measures. We conducted a literature search on this topic and, as a first step, summarized the design and main findings of these empirical studies with a particular focus on the heterogeneity between studies. Furthermore, we present our suggestions for future VR-based NF investigations. Based on the literature review, we also discuss the advantages and disadvantages of using VR as a feedback modality. In the final section, general ethical considerations in the use of commercial VR-based NF systems are addressed.

## Review of VR-Based NF Studies

**Fig. 2:**
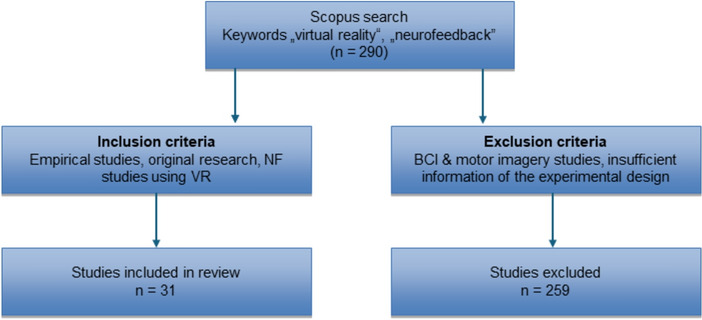
Flow chart of literature search

The aim of this review was to summarize empirical articles that investigated the effects of VR-based NF training on various outcome measures. Note that VR-based feedback is also used in brain-computer interface (BCI) applications. While one of the primary aims of BCI research is to control external devices (e.g., wheelchairs, prosthetics) with specific brain signals (Wolpaw, 2012), the aim of NF training is to modulate one’s own brain activity in a pre-defined direction for the improvement of cognitive, motor or affective functions (Gruzelier, 2013, 2014; Jeunet et al., 2019). For a review of VR-based BCI applications, please see for instance Leeb and Pérez-Marcos, (2020), Wen et al. (2021), or Lotte et al., (2014). In the present review, we focus on EEG-based VR NF studies. Therefore, we conducted a Scopus search (May 2024) for the keywords “neurofeedback” AND “virtual reality”. Of the 290 documents resulting from this search, we filtered for empirical studies which used VR-based feedback during NF training and provided sufficient information on the experimental design (description of the sample, NF protocol, experimental conditions, VR system and feedback, outcome measures, and main outcomes). Ultimately, 31 studies that met these criteria remained in the review (see Fig. 2).

Table 1 summarizes the design and results of the included VR-based NF studies. The included studies are heterogeneous in terms of methodology, design and outcome measures. In the following, we will summarize the differences and similarities of the VR-based NF studies and provide suggestions for future studies.

**Table 1:** Summary of VR-based NF studies

Authors	Sample	Feedback frequencies & electrodes	Number of sessions	Conditions	VR system	VR feedback	Outcome measures	Main outcome
Abdessalem et al. (2018)	20 healthy subjects (10 female)	NA (emotive system)	1	3D VR-feedback	Fove VR	Driving an ambulance with a sick person on its way to hospital Difficulty adapts related to EEG frustration levels	Emotion (Frustration & Excitement) for game experience	Game difficulty & speed adapted alongside frustration/excitement level
Ai et al. (2021)	5 healthy subjects (lab students)	NA	1	3 interaction methods: 3D buttons, speech instruction, NF	NA	3D virtual reality animal environment, interaction with virtual animals in zoo	User experience	It is possible to interact with virtual animals in virtual Zoo Therapy
Arpaia et al. (2023a)	3 healthy subjects	Beta 20–34 Hz, FCz-CPz	3	2D visual feedback vs. 3D VR-feedback	HMD	Negative International Affective Picture System images were presented	Emotional regulation capacity, NF training performance	Beta band power during NF training remained below the baseline, as expected. No difference between 2 and 3D VR NF
Arpaia et al. (2023b)	3 subjects one of which (> 65 years) with onsets of neuromotor slowdowns	High-Beta 20–34 Hz, bipolar channel FCz-CPz	6	2D NF vs. 3D VR NF	HMD	Negative International Affective Picture System images were presented in a virtual office room; room lighting color was manipulated and thermometer	Average mu and beta absolute power spectral densities in right sensorimotor area	Decrease of mu and beta powers in VR compared to 2D
Berger and Davelaar (2018)	22 healthy subjects	Alpha 8–12 Hz, FP2	5	2D visual feedback vs. 3D VR-feedback	HMD	Virtual room, blue vase floated	NF training performance, attentional control	Relationship between NF learning rate and changes on deployment of attentional control. No difference between 2 and 3D VR NF
Berger et al. (2021)	68 healthy subjects	SMR 12–15 Hz, Theta 4–7 Hz, Beta 16–30 Hz, Cz	1	2D visual feedback vs. 3D VR-feedback	HMD	Roll a ball along a predefined path in a virtual forest environment	User experience	Women show stronger cybersickness symptoms and more negative user experience than men
Berger et al. (2022)	61 healthy subjects	SMR 12–15 Hz, Theta 4–7 Hz, Beta 16–30 Hz, Cz	1	2D visual feedback vs. 3D VR-feedback; Real vs. sham feedback	HMD	Roll a ball along a predefined path in a virtual forest environment	NF training performance	NF performance higher in 3D VR condition than in 2D condition, but NF performance comparable between real and sham condition
Berger et al. (2024)	41 healthy subjects	SMR 12–15 Hz, Theta 4–8 Hz, Beta 16–30 Hz, Cz	1	Pseudo brain stimulation vs. no pseudo brain stimulation	HMD	Roll a ball along a predefined path in a virtual forest environment	NF training performance, cybersickness	Women show stronger cybersickness symptoms and worse NF training performance than men
Chen et al. (2024)	30 healthy subjects	Alpha 8–12 Hz, AF7, AF8	1	Out-of-body (OBE) vs. in-body (IBE) condition	HMD	3D videos showing different conditions, speed, brightness, and color saturation of the videos changed	Changes in different EEG frequency bands, body experience	Body experiences stronger during out-of-body condition. Theta lower during the OBE than the IBE, difference in alpha and beta across electrodes varied between conditions
Cho et al. (2004)	28 subjects, who had committed crimes and had been isolated in a reformatory, aged 14–18 years	Beta wave ratio 15–18 Hz, Cz	8	2D visual feedback vs. 3D VR-feedback vs. waiting control group	HMD	Virtual classroom, dinosaur egg and puzzle	Inattention and impulsiveness	NF lead to improvements in inattention and impulsiveness compared to waiting list control group. No difference between 2 and 3D VR NF
Cohen et al. (2016)	30 healthy subjects	Amyg-EFP model, Pz	2	2D visual feedback vs. 3D VR-feedback	Not specified	Virtual hospital waiting room that may be either agitated or relaxed	NF training performance and transfer to other situations, user experience	3D VR NF produced higher effect size, showed better sustainability and better transferability to a new unfamiliar interface compared to 2D NF. Interest/enjoyment, usefulness, relatedness, and perceived competence was higher in 3D VR NF than in 2D NF condition
Connelly et al. (2023)	15 patients with migraine, aged 10–17 years	Delta, theta, low-alpha, high-alpha, low-beta, high-beta, low-gamma, and mid-gamma, FP1	NA	3D VR without feedback vs. 3D VR-feedback vs. AR-feedback	HMD	Choice of different scenes, e.g., underwater scene, forest and waterfall scene, beach scene, snow and streaming water scene, waterfall scene, rainfall scene. VR changed based on brainwave data (e.g., rain stopped)	User experience	No differences in side effects between 3D VR with and without NF and AR-feedback. Side effects were generally mild. Acceptability questionnaire scores exceeded a minimum threshold, 3D VR conditions with and without NF were preferred over AR-feedback
D’Errico et al. (2023)	3 male automation engineering students	High-beta power 20–34 Hz, Fz	1	VR-NF with audio and visual feedback	HMD	Change audio tunes and shapes to reduce high beta and anxiety	Usability	Participants rated the set-up with acceptable usability levels
Gu and Frasson (2017)	6 healthy subjects	Feedback frequency not specified, F3, F7, F3, FC5, T7, P7, O1, O2, P8, T8, FC6, F4, F8, and AF42	8	3D VR-feedback	HMD	3 relaxing environments: Seaside, Japanese Garden and waterfall	Subjective relaxation	Decreases in anxiety and depression, decrease in Time Interval to Relaxation and an increase in the maximum Meditation Score
Guedj et al. (2023)	4 healthy children	Theta/beta ratio 4–7.5 Hz/13–19 Hz, Fz	8	3D VR-feedback	VR cave	Virtual classroom	NF training performance, user experience, attentional performance	Stable attentional performance across multiple sessions, which might indicate sustained level of motivation. 3 out of 4 children demonstrated a decrease trend in their TBR during the later stage of training. All children reported enjoyment of the study
Gruzelier et al. (2010)	15 drama students	SMR 12–15 Hz, Beta 15–21 Hz, Theta 4–8 Hz, Cz	7–10	2D visual feedback vs. 3D VR-feedback vs. no training comparison group	VR cave	Lighting level of virtual auditorium increased or decreased with simultaneous changes in intrusive audience noise	Acting performance, NF training performance, user experience	3D VR NF group and 2D NF group increased target EEG frequency across sessions, 3D VR group reached peak level earlier than 2D group. Stronger improvement in acting performance in 3D VR group than in 2D group. 3D VR group also reported highest flow experience
Han et al. (2019)	30 healthy subjects	Alpha 8–13 Hz, FP1		VR vs. real environment	Laptop screen	Control speed of a racing car increasing proportional to concentration level	Band power of alpha frequency range & performance in a Stroop task	Comparable alpha frequency range and cognitive test score between both groups. NF in virtual environment led to higher training effects compared to real environment
Juliano et al. (2020)	12 healthy subjects	Broad activity in alpha and beta bands 8–24 Hz, C1, C3, and CP1		2D visual feedback vs. 3D VR-feedback	HMD	Moving virtual hand, hitting a ball at a beach	NF training performance and user experience	NF training performance was similar between conditions. Higher levels of embodiment in 3D VR condition. Levels of embodiment positively correlated with NF performance only in 3D VR condition. No differences in cybersickness symptoms between groups
Kober et al. (2016)	9 stroke patients	SMR 12–15 Hz, Theta 4–7 Hz, Beta 13–21 Hz, Cz	5–10	2D visual feedback vs. 3D VR-feedback	Shutter glasses, 3D monitor	Human body, brain changed colors	NF training performance and user experience	NF training performance comparable between conditions. Feeling of control and higher in 3D VR condition than in 2D condition. Higher incompetence fear and lower mastery confidence in 3D VR condition than in 2D condition
Kober et al. (2017)	24 healthy older individuals and 2 stroke patients	SMR 12–15 Hz, Theta 4–7 Hz, Beta 13–21 Hz, Cz	10	2D visual feedback vs. 3D VR-feedback	Shutter glasses, 3D monitor	Human body, brain changed colors	NF training performance and user experience	NF training performance higher in 3D VR condition than in 2D condition. In the 3D VR condition, confidence was lower and fear was higher in patients than in healthy controls
Lu et al. (2022)	18 healthy subjects	Alpha band 8–13 Hz, POz	2 (CG and EG within group)	NF (experimental group) vs. no NF (control group) during tracking task	HMD	Track colored dots in a moving dot-cloud, getting more or less transparent depending on level of focus	NF performance (alpha power)	Alpha band power decreases in the NF group compared to the control group, task performance is improved by 6.44% in the NF group
Orakpo et al. (2021)	1 patient with chronic pain	0.15 mHz at T3–T4, 0.175 mHz at T4–P4	20	3D VR-feedback	HMD	Cygnet Software for the NF functions, all provided by BeeMedic, no more detailed description	Pain	40% decrease in pain, activities of daily living improved by 40%, independent activities of daily living improved by 50%, pain-related anxiety improved by 40% after NF training. No prominent changes in sleep deprivation, pain-related fatigue, or PTSD flashbacks
Orakpo et al. (2022)	1 patient with insomnia and chronic pain	Infra-low frequency, T4—P4	20	3D VR-feedback	NA	Various games and videos	Pain, insomnia	The patient showed significant improvement in both pain (60%) and insomnia (70%)
Rolbiecki et al. (2024)	30 cancer patients	NA	2	VR only vs. VR + NF	HMD	Nature based videos, EEG was data displayed with a small orb of light	Cancer symptoms	VR + NF group showed improvements in anxiety and improved pain, VR only group showed improvements in pain and depression
Salminen et al. (2023)	43 healthy university students	Theta 4–6 Hz, average of F3 and F4, Alpha 8–12 Hz, average of F3, F4, C3, C4, P3, P4	1	NF + VR with meditation or body scan, no NF + VR with meditation or body scan, no NF + Computer screen with meditation or body scan	HMD	Human body showing parts for targeted attention or point focus meditation with floating spheres. Controlling movements and opaqueness in both paradigms	Presence, meditation depth, brain activation	Higher feeling of presence in VR condition and when NF was used. More meditation depth in VR conditions
Tarrant and Cope (2018)	4 firefighters	Gamma 30–44 Hz, AF7-AF8	1	3D VR-feedback	HMD	360-degree video photography with an additional blue and red line	Mood, gamma asymmetry	Gamma asymmetry shift after NF training. Mood changed after NF training
Tarrant et al. (2022)	100 healthcare workers	High Beta 18–29.75 Hz, FP1 & FP2	1	3D VR-feedback meditation vs. audio-only meditation	HMD	Glowing ball that moves up and own in relation to changes in EEG amplitude with beach scenery in the background	Anger, depression, tension, fatigue, vigor, happiness, calmness, confusion	Both groups showed a similar and significant decrease in anger, tension, and depression. Vigor, fatigue, and confusion decreased while calmness and happiness increased in the 3D VR NF group
Vourvopoulos et al. (2019)	4 chronic stroke patients	Broad activity in alpha and beta bands 8–24 Hz, C3, C4	8	3D VR-feedback	HMD	Moving virtual hand, hitting a ball at a beach	NF performance, user experience, muscle movement	NF training performance increased in 1 out of 4 patients across NF sessions. Patients with more severe motor impairments may benefit the most from EEG-based NF, while patients with more mild impairments may benefit more from EMG-based feedback. No significant changes in cybersickness
Yamin et al. (2017)	2 epilepsy patients	Gamma, 25–35 Hz and 65–85 Hz, amygdala depth electrode	1–2	3D VR-feedback	Screen	Controlling virtual crowd in virtual room	NF performance	One patient successfully down-regulated gamma in 2 out of 8 runs, the other patient showed no significant changes in gamma power. Down-regulation of the higher gamma band (65–85 Hz) was more prominent than the lower band (25–35 Hz) in both patients. Modulation in power was restricted to the gamma band
Yang et al. (2019)	60 healthy subjects	NA	1	3D VR reminder feedback vs. 3D VR encouraging feedback vs. no feedback	HMD	Auditory feedback during VR creativity task	Creative performance	Reminder feedback led to higher-quality creative products than other conditions. 3D VR feedback affected attention and flow
Yu et al. (2023)	8 healthy college students	Theta 3–7 Hz, Alpha 8–13 Hz, Beta 14–29 Hz, Gamma 30–47 Hz	6	8 3D VR scenarios (4 positive and 4 negative)	HMD	Cognitive reappraisal to reduce negative emotions—should change VR scenery from negative to positive	Emotion regulation, self-esteem, hospital anxiety, depression	Increase of cognitive reappraisal, expressive suppression scores were lower, increase in overall emotion regulation ability, reduction of impulse, decrease of strategies, higher self-worth after the training

*2D* Two-dimensional, *3D* Three-dimensional, *CG* Control group, *EG* Experimental group, *HMD *Head-mounted display, *NA* Not apparent, *NF* Neurofeedback, *SMR* Sensorimotor rhythm, *VR* Virtual Reality

### Sample

The sample sizes of the studies included in this review range from 1 (Orakpo et al., 2021) to 100 (Table 1) (Tarrant et al., 2022). Sample sizes are largely not justified or determined by means of a priori power analysis, with the exception of three studies (Berger et al., 2022, 2024; Tarrant et al., 2022). These studies reported an a priori sample size calculation with G*Power (Faul et al., 2007). Some studies were of exploratory nature or pilot studies, and certain clinical studies were case reports, explaining the lack of sample size justifications in those cases, yet this does not account for all the studies. Clinical samples include migraine, stroke, chronic pain, epilepsy, or cancer patients. Most studies investigated non-clinical individuals. Relatively small and unjustified sample sizes pose problems with regards to statistical validity and generalizability of the results.

### Feedback Frequencies & Electrodes

As is customary in NF studies, different EEG feedback frequencies and feedback electrodes are selected depending on the primary objective and outcome measurement of the NF training (Table 1) (Gruzelier, 2014). In six studies, the specific feedback parameters were not fully specified. It is generally recommended to specify the feedback parameters precisely, in order to enable reproducibility of the NF training paradigm as well as to increase interpretability of training results (Ros et al., 2020).

### Number of Sessions

The number of sessions range from 1 to 20 between the different studies (e.g., Berger et al., 2022; Orakpo et al., 2021) (Table 1). Generally, the number of sessions depends on the aim of the study. If the aim of an NF training paradigm is to improve cognitive or behavioral functions, repeated training sessions are necessary. For instance, it is suggested that NF training aimed to reduce symptoms of attention deficit hyperactivity disorder (ADHD), requires multiple training sessions (e.g., 30–40 sessions) over several weeks or months (Arns et al., 2014; Enriquez-Geppert et al., 2019). If the primary aim of the training is to investigate the effects of VR-based feedback, e.g., on user experience, a single session can already suffice. However, in order to demonstrate the consistency of these effects, repeated training sessions are advantageous. Unspecific VR-related effects, such as excitement (Ali et al., 2014) and novelty factors (Wow-effect) may disappear over repeated training sessions.

### Conditions

Many studies include only one condition, namely a 3D VR-feedback condition. Many studies lack control conditions or control groups (Table 1). Without the inclusion of an appropriate control condition (e.g., 2D feedback condition, sham feedback or feedback of another EEG signal) it is difficult to draw robust conclusions concerning treatment efficacy as well as specific and unspecific effects (e.g., placebo effects) of VR-based NF training (Ros et al., 2020; Thibault et al., 2016, 2017). Adherence to the guidelines proposed by Ros et al., (2020) on designing and reporting NF studies is a welcome perspective for future studies. Berger et al., (2022) compared 3D VR-feedback and 2D feedback as well as real and sham feedback. They found that 3D VR-feedback led to an improved NF performance compared to 2D feedback. However, this was the case for real as well as for sham feedback indicating rather nonspecific effects of VR on NF performance (Berger et al., 2022). This study shows that only the inclusion of suitable control conditions can reveal such nonspecific effects.

The majority of studies including control conditions compared traditional 2D visual feedback screens with 3D VR-feedback (e.g., Arpaia et al., 2023a; Berger & Davelaar, 2018; Berger et al., 2022; Cohen et al., 2016; Gruzelier et al., 2010). These studies provide an indication of whether more complex and visually enriched 3D VR feedback can lead to different NF-related results, when being compared to simple 2D feedback on a computer screen (e.g., moving bars). Arguably, studies in this category have the weakness of unbalanced motivation across groups, given that a 3D VR environment is usually more attractive than their 2D counterparts. Study designs limited to one or a few training sessions are expected to produce the most pronounced effects favoring the 3D VR group. The findings of these studies are mixed. Some studies found beneficial effects of 3D VR-feedback on NF training performance (Berger et al., 2022; Cohen et al., 2016; Kober et al., 2017), whereas others did not (Arpaia et al., 2023a; Berger & Davelaar, 2018; Gruzelier et al., 2010; Juliano et al., 2020; Kober et al., 2016). However, studies that did not find difference in NF training performance between 3D and 2D conditions found differences in subjective user experience such as affective states or embodiment (Juliano et al., 2020; Kober et al., 2016). Although it seems reasonable to compare conventional 2D feedback with 3D VR-feedback to identify potential advantages and disadvantages of VR-feedback, such a comparison must be made under certain considerations. The comparison between VR-based and traditional 2D feedback is affected by a number of visual features that differ between these two types of visual presentation (e.g., field of view, visual richness, brightness of the screen, screen size, etc.). Such visual features can have VR-unspecific effects on brain activation, independent of the NF training itself. For instance, screen brightness may affect Alpha activity (Park et al., 2013), meaning if brightness levels of the VR screen and the 2D computer screen differ, the interpretability of task-related changes in alpha power is impaired. It becomes more difficult to determine whether differences in Alpha power during NF training are caused by differences in e.g., the relaxation level achieved through NF or by differences in the visual input itself. In this context, we advise the inclusion of passive viewing conditions as control conditions, i.e., where participants see the moving 3D VR and 2D visual input without NF, to control for such unspecific visual effects.

### VR System

Comparing the results from different VR-based NF studies is further complicated by the differences in the used VR systems and technologies (Table 1). While most studies use head-mounted display (HMD) systems (e.g., Berger et al., 2022; Vourvopoulos et al., 2019), some studies utilized 3D monitors (Kober et al., 2016, 2017), cave-systems in which the participants are surrounded by projection walls (Gruzelier et al., 2010), or even laptop screens (Han et al., 2019). 3D VR systems with a stereoscopic stimulus presentation might be more immersive than screen systems with monoscopic view, leading to differences in presence experience in VR (Freeman et al., 1999; IJsselsteijn et al., 2001; Kober et al., 2012), which can also affect participants’ behavior, performance, as well as brain activation (Kober et al., 2012). In this context, Gruzelier et al., (2010) reported higher levels of flow experience in a 3D VR-cave condition compared to a 2D condition. This could have an impact on the user experience and the training outcome. However, it is difficult to draw a general conclusion about the effectiveness of VR-based NF training given the heterogeneity of VR systems used in the literature.

### VR Feedback

There is also a wide variety of different VR feedback scenarios (Table 1). Some examples include moving a virtual ball through a forest (Berger et al., 2022), interacting with animals in a virtual zoo (Ai et al., 2021), or controlling a virtual crowd (Yamin et al., 2017). This aspect hampers the comparability between studies even more, since such feedback scenarios usually also differ in their level of task difficulty or user engagement. More research is needed concerning the effects of visual rich and complex VR feedback on NF performance and related outcome measures.

### Outcome Measures

The reviewed studies also largely focus on different outcome measures (Table 1). Not all studies report on the effects of 3D VR-feedback on NF training performance. Especially patient studies mainly focus on changes in clinical symptoms (e.g., Orakpo et al., 2021; Rolbiecki et al., 2024). Studies in healthy individuals more often focus on user experience (Ai et al., 2021; Berger et al., 2021; Connelly et al., 2023). We would generally recommend to always also report NF performance, as additional knowledge about potential correlations between NF performance, user experience or changes in clinical symptoms is also highly relevant for assessing the effectiveness and specificity of NF.

### General Recommendations for Future Studies

We advise that future studies should adhere to established NF standards with regard to the design and reporting of NF studies (nf-CRED checklist by Ros et al., 2020). Precise and accurate reporting is an important issue to improve the reproducibility as well as interpretability of studies. In our review, we encountered several issues in determining the minimum design parameters of individual studies, as shown in Table 1. For instance, NF parameters such as exact feedback frequency or electrode location were not always specified. To reduce publication bias and to improve the quality of VR-based NF studies, it is imperative to describe feedback design, parameters, online-data processing etc. in detail as suggested by Ros et al., (2020).

## Why should VR be Used as a Feedback Method?

### Increase Positive Affect with More Entertaining, Game-Like VR Feedback

One of the main reasons to use 3D VR-feedback is to increase positive affect and motivation with more game-like and entertaining VR scenarios (Kober et al., 2018). Motivation can influence NF training performance (Kleih et al., 2010; Kleih-Dahms & Botrel, 2023). NF training often requires multiple repeated training sessions over weeks or even months (Arns et al., 2009). To keep users motivated and increase adherence to training, more interesting and motivating feedback may improve the overall experience. It is assumed that 3D VR-feedback is more motivating and entertaining. However, to investigate this in more detail, it is necessary to assess the subjective user experience during VR-based NF training and compare it with, e.g., traditional 2D feedback or passive viewing conditions. Previous studies investigating user experience generally report more positive user experience during 3D VR-feedback compared to 2D feedback. For instance, Cohen et al. (2016) found that interest/enjoyment, usefulness, relatedness, and perceived competence was higher in a 3D VR-feedback condition than in a 2D NF condition. Guedj et al. (2023) reported a high level of enjoyment of children using a 3D VR-feedback. However, studies investigating samples of higher age or clinical samples, which are presumably not as familiar with VR technology, report on negative affective reactions such as increased fear in 3D VR-feedback conditions (Kober et al., 2016, 2017). Despite these isolated negative reports, the majority of studies investigating user experience report positive effects of 3D VR-feedback on subjective user experience.

As mentioned earlier, it is possible that such motivating and entertaining effects of VR-based NF training may disappear over time, when the novelty effect of VR-feedback diminishes. Therefore, it is of high relevance to perform more VR-based NF studies over a longer time span.

### Multimodal Feedback

VR also enables multimodal feedback, for instance the combination of visual, auditory as well as tactile feedback. For instance, Gruzelier et al. (2010) used multimodal VR-feedback combining visual and auditory feedback. In a virtual auditorium the lighting level increased or decreased depending on changes in SMR activity, but also the intrusive audience noise increased when non-target EEG frequencies increased (Theta, Beta). Although examples of multimodal feedback integration exist, the majority of VR-based NF studies used unimodal (visual) feedback (Table 1).

Multimodal feedback enables a more user-centered design of NF applications (Kleih & Kübler, 2018; Kübler et al., 2014). Some NF users might prefer visual feedback, others might prefer auditory feedback or combined feedback. VR feedback can be adapted individually such that participants feel comfortable, reach a state of flow, can concentrate well and feel immersed in the VR. User-centered design approaches involve designing assistive technology with the target population involved in the active research and development process (Botrel et al., 2015; Münßinger et al., 2010). Studies on the effects of a user-centered design showed that adapting the NF paradigm to the user’s needs (e.g., by adjusting the amount of presented information), can increase training performance and it enables end-users to better learn NF/BCI operation (Kleih et al., 2016).

### Transfer to Real World

An important issue in NF studies is whether NF users can transfer the mental state they reach during NF training to other contexts without real-time feedback of their own brain activation. For instance, children with attention deficit hyperactivity disorder (ADHD) should be able to produce the mental state, which they reach during NF training when they, e.g., decrease their Theta/Beta ratio, during school as well. In this context, there is the possibility to simulate a virtual classroom, so that children can train this transfer in VR.

Generally, NF studies providing traditional 2D feedback of slow cortical potentials (SCP) included such transfer trials (e.g., Strehl et al., 2006). These studies either included transfer trials without contingent feedback in their NF protocols or gave home-work, where the participants should try to reach the mental state as it was reached during NF training but without any EEG measurement (Gevensleben et al., 2010). Hence, it is not clear whether the participants could reproduce the mental and neuronal state reached during NF at home. A study by Cohen et al. (2016) is one of the rare examples in this context investigating such a transfer to new situations. They investigated the transferability of NF control to a new unfamiliar interface. NF training was performed in a 3D virtual hospital waiting room or in a 2D condition (thermometer). Afterwards, the NF training was performed in another scene consisting of a 2D moving skateboard interface. The 3D VR-feedback led to a better transfer to the new feedback interface compared to 2D NF (Cohen et al., 2016). The study by Cohen et al., (2016) therefore suggests that VR-based feedback scenarios could promote the transfer of the mental states achieved during NF training to other contexts. Future VR-feedback studies should make greater use of the possibility of creating transfer scenarios in VR, as the ability to design ecologically valid virtual environments is a major strength of VR compared to conventional feedback modalities (Parsons, 2015).

## Contraindications

Using VR as feedback modality might not only have positive effects. It is of high relevance to investigate and report also possible adverse effects and to identify suitable target groups and groups of NF users, for whom VR-based feedback might not be beneficiary.

### Cybersickness

Cybersickness is a well-known issue in the field of VR and describes the occurrence of sickness symptoms during the interaction with virtual environments similar to motion sickness. Symptoms that can occur are dizziness, nausea, oculomotor problems and disorientation. Studies report that in general up to 80% of all VR users can be affected (Kim et al., 2005; Rebenitsch & Owen, 2016).

There are different assumptions describing the reasons behind the occurrence of cybersickness. While biological causes are not clearly described yet (Davis et al., 2014; Kim et al., 2005; Rebenitsch & Owen, 2016), different theories exist. The most common theory is the “Sensory Mismatch Theory”, describing the discrepancy of how inputs are processed by different senses. Another common theory relates to postural instability, explaining emerging sickness by posture adaptations next to existing and contradicting gravitational forces. Finally, the “Rest Frame Theory” describes the effects of discrepancy to whether directions in VR relate to directions in the real world. The “Poison Theory” in the domain of sickness research, however, is not applied to cybersickness (Kim et al., 2005; Rebenitsch & Owen, 2016). Considering this theoretical background, sickness can occur due to different factors in a virtual environment, whereby not all of them are relevant for NF training processes. On a more general note, some of them include issues with the presentation display and technology, such as bad frame rates and latencies. However, due to advancements in the state-of-the-art technology, providing for instance higher frame rates and shorter latencies, these issues are becoming less of a concern. Other issues could be errors with position tracking and are especially relevant when using devices including sensor boxes, which are less relevant for wireless systems with integrated position tracking sensors (LaViola, 2000). Also, as NF users are primarily sitting motionless during training, position tracking issues are less of a problem. What is especially problematic however, are virtual movements. While VR paradigms for NF do not necessarily incorporate movement of the environment, there is the potential to make environments more dynamic. Some researchers incorporated moving environments into the NF training paradigm (Abdessalem et al., 2018; Berger et al., 2024). Most of the studies mentioned here, however, rely on static paradigms (e.g., Juliano et al., 2020; Yang et al., 2019). Besides nausea symptoms, oculomotor problems also account for cybersickness. VR-exposure can lead to eye strain and eye fatigue (Iskander et al., 2018), making it difficult to focus, independently from whether the VR environment is moving or not.

Cybersickness symptoms usually disappear within several minutes of VR usage (Ames et al., 2005). Nevertheless, it affects user experience and could therefore negatively influence NF performance. The most commonly used questionnaire to control for symptoms of cybersickness is the Simulator Sickness Questionnaire (SSQ) (Kennedy et al., 1993), and there already exists a VR-specific version (VRSQ) (Kim et al., 2018). Still, cybersickness is not always reported in VR-based NF studies (e.g., Berger & Davelaar, 2018; Gruzelier et al., 2010; Yang et al., 2019; Yu et al., 2023). In some studies, participants were screened beforehand in order to exclude those prone to develop motion sickness (Berger & Davelaar, 2018; Tarrant et al., 2022). Controlling for cybersickness is encouraged to better evaluate the best design-specific decisions in VR-based NF. This is especially relevant for the application in vulnerable groups such as clinical patients. In a set-up with a few consecutive short NF runs, there was no overall associations between cybersickness and NF performance (Berger et al., 2024). However, women were more severely affected by sickness symptoms than men and showed worse NF performance. Hence, there still could be an association between sickness symptoms in VR and NF performance (Berger et al., 2024). Women are more frequently and more severely affected by cybersickness symptoms in VR than men (Chang et al., 2020). One explanation for this could be that the interpupillary distance settings of HMDs can in many cases not be sufficiently adapted to women’s anatomy, which could result in the issue that visual focus points not properly align. In addition to sex, age and prior VR experience can also influence symptoms of cybersickness (Chang et al., 2020; Kourtesis et al., 2024; Paillard et al., 2013). This should be considered when designing VR-based feedback for different user groups.

The majority of studies assessing cybersickness during VR-based NF found no differences in cybersickness between computer screen-based training and 3D VR-feedback (Berger et al., 2024; Juliano et al., 2020; Kober et al., 2017) or changes over time (Vourvopoulos et al., 2019). Kober et al., (2017) even report a decrease in sickness symptoms over time. In one study most participants reported “none” to “slight” cybersickness symptoms (Connelly et al., 2023). Others reported an increase in cybersickness after performing VR-NF training, however the more immersive 3D VR environment did not elicit more sickness than the less immersive 2D VR environment (Berger et al., 2021). The few studies that investigated cybersickness in VR-based NF studies therefore found no cybersickness-inducing effects of VR feedback. However, this does not mean that cybersickness should be neglected in VR-based NF studies. We strongly encourage monitoring cybersickness during VR-NF training sessions via questionnaires and objective measures such as heart rate or skin conductance, not only to gain more insight into the relationship between VR and cybersickness, but also possible correlates with NF performance.

In general, cybersickness can be rather high in VR studies (Kim et al., 2005; Rebenitsch & Owen, 2016), but 3D VR-feedback studies do not report strong cybersickness effects during NF training. The reason for that might be that the 3D VR-feedback paradigms used in prior NF studies were not as immersive, e.g., when using static feedback such as a virtual human body (Kober et al., 2016, 2017; Salminen et al., 2023). With more interaction and active movement in VR, cybersickness may be more pronounced or affect NF performance to a greater extent (Berger et al., 2024). So far, no reports of an association between cybersickness and NF performance could be found.

### Age

Because the market for affordable VR headsets is growing, many people already have a VR system at home, using it primarily for entertainment. Mainly people under the age of 50 have experience with VR whereas people older than 50 years are barely familiar with VR technology (Eg & Raaen, 2024). This age discrepancy must be considered when using e.g., VR-based NF with older individuals. Studies generally show that elderly users navigate and experience immersion and levels of presence in virtual environments differently (Kober, 2014). Moreover, people above 40 years are more prone to develop symptoms of cybersickness (Saredakis et al., 2020). NF studies by Kober et al., (2016) and Kober et al., (2017) showed that older NF users show higher levels of fear during NF training in 3D VR-feedback conditions compared to 2D conditions, and stroke patients show higher levels of fear during 3D VR-feedback compared to healthy older individuals (Kober et al., 2017). This might be due to a lower technology usage in elderly compared to younger individuals. Hence, the effects of VR-based NF could depend on the age of the users.

### Cognitive Overload

As mentioned above, VR offers the possibility of presenting multi-modal feedback. Many HMD systems for instance support headphones or have integrated loudspeakers for auditory stimulation. Yet, more complex sensory stimulation can potentially overstrain cognitive resources. Too much sensory stimulation in VR can also be a distraction from the actual task at hand. NF studies that investigated the mental strategies used by participants during NF training showed that, e.g., for SMR-based NF training, no specific mental strategy seemed to be superior over others and using highly vivid task-irrelevant mental strategies was even counterproductive for NF performance (Kober et al., 2013). There is evidence from the educational context, that the inclusion of game elements in learning material specifically for the improvement of learning outcome, not always shows positive results. It was shown that including too many game elements in a learning task, which capture attention but are not directly necessary to fulfill the task, are more distracting than helpful and can interfere with the learning process (e.g., Rey, 2012, 2014). Distracting task-irrelevant information might lead to poorer learning outcomes caused by increased cognitive/mental load (Schrader & Bastiaens, 2012). Using highly vivid and realistic VR scenarios for NF could also increase task-irrelevant thoughts or cognitive overload interfering with NF performance. Future studies should address this issue by comparing for instance visually rich VR-feedback with simpler VR-feedback as well as assessing cognitive load, e.g., by using questionnaires.

## Where are we going?

VR-based feedback seems to be promising but more research is needed to determine positive and negative effects of VR-based NF training.

International companies are working on brain implants to control for instance a smartphone or virtual environments. Others produce and sell affordable high quality VR headsets, thereby increasing the availability of VR systems in the general population. Some experts predict a future, in which interacting in a metaverse, which is a network of VRs in which individuals interact, will become even more important not only for entertainment but also for work, school, rehabilitation, and even therapy (Keshner et al., 2019; Park et al., 2019; Radianti et al., 2020). Affordable and easy-to-use EEG headsets as well as VR headsets with integrated EEG electrodes are already available (Paek et al., 2021). Hence, controlling VRs or a metaverse with brain signals in everyday life situations outside the lab could be a part of the foreseeable future. Some users are already adapting commercial video games to be controlled by their own brain activity recorded using commercial EEG recording technology. On the one hand, the development and progress of home-based VR-NF interfaces could facilitate home-based training and rehabilitation (Autenrieth et al., 2022; Kober et al., 2019). On the other hand, the access of large companies providing such VR-based NF systems to brain data raises several concerns about personal data protection (Genser et al., 2024). In this context, the expectations towards the new technologies—also known as sociotechnical imaginary (Williamson, 2017)—play an important role.

As discussed above, the high degree of immersion created by modern VR technologies has the potential to boost motivation to undergo laborious interventions, which otherwise would not be sufficiently motivating to certain clinical groups. In this regard, VR can be a game changer for NF treatment. However, the highly captivating nature of VR also has negative aspects to it. One of them regards the gray zone between NF as a health intervention and NF as a form of entertainment and self-fulfillment. As a health intervention, NF should be understood as a clinical therapeutical practice to treat disorders and clinical medical/psychological symptoms. As entertainment and self-fulfillment, NF acquires a completely different character and should be seen as an enhancement practice (Paek et al., 2021). While the framing of NF is not clear and the disparity between scientific evidence and practice remains large, misrepresentation of services and misleading advertising can be expected to remain common practice among NF providers (e.g., Wexler et al., 2020).

Typically, the amount of supervision dedicated to the practice of NF under the label of entertainment is much lower than as a therapeutical practice. This applies not only to VR-based NF training, but to NF training in general. Consequentially, users feel safe to use NF for prolonged periods of time. Although there are hints in the literature that NF can have certain adverse side-effects to users (Kober et al., 2015), these occur too rarely to document their frequency and intensity in different populations. Existing studies predominantly provided post-hoc debriefing of participants which is generally limited to superficial non-cognitive symptoms such as skin irritations or headaches. Adverse effects of NF are therefore poorly understood and frequently dismissed without proper investigation. Cognitive side-effects of NF training also should be expected, as it is the case with every effective form of treatment (Thibault & Raz, 2017). The prolonged practice of NF with the aim of enhancement may lead to undesired effects of plasticity (Ikoma et al., 2023; Lloyd & Dayan, 2023). VR-based NF for the purposes of personal enhancement and entertainment could promote increased usage which could in turn lead to adverse side-effects. However, cognitive testing alongside NF training is mostly missing, as well as longitudinal studies in this field.

As has been discussed previously (Kober et al., 2023), neuroenchantment (Ali et al., 2014) reduces critical thinking during the evaluation of the capabilities and limitations of neuro-technologies. As demonstrated by Olson and colleagues (Olson & Raz, 2021; Olson et al., 2016), it is easy to manipulate study participants into expecting high levels of efficacy even from mock neuro-technologies. Participants would also believe a machine’s conclusions about their preferences and attitudes over their own conclusions, making them highly susceptible to manipulation. Moreover, VR has a strong potential to exacerbate existing problems with scientific rigor in NF research (e.g., the severe flaws of QEEG, Wood et al., 2024). The multimodal appeal of the VR device may cast a shadow over flaws in study design that are still common in the NF research literature (Thibault et al., 2017) and for which quality control guidelines have been developed (Ros et al., 2020).

The surge of VR can also contribute to increasing user acceptance of privacy intrusions by companies and undermine the proper application of data protection law. In a recently published report of the Neurorights Foundation, 30 consumer neurotechnology devices have been assessed concerning the topic of privacy (Genser et al., 2024). It was reported that over 50% of the reviewed companies state in their policies the provision of sharing data with third parties. Almost 97% of all the reviewed neurotechnology companies have access to neural data of the consumers. Considering that consumer devices are getting increasingly popular, the issue of personal data protection is becoming even more relevant, raising the necessity for legal regulation.

The use of highly immersive and entertaining VR-feedback in NF applications should not distract from privacy and data protection issues regarding data security as well as “neurorights”. The development and application of home-based VR-NF systems has the potential to provide cost effective and easy-to-use therapy and training environments, yet ethical considerations, data security and privacy should not be neglected in this regard.

## Data Availability

No datasets were generated or analysed during the current study.
